# Face and voice identity matching accuracy is not improved by multimodal identity information

**DOI:** 10.1111/bjop.12757

**Published:** 2024-12-17

**Authors:** Harriet M. J. Smith, Kay L. Ritchie, Thom S. Baguley, Nadine Lavan

**Affiliations:** ^1^ NTU Psychology Nottingham Trent University Nottingham UK; ^2^ School of Psychology University of Lincoln Lincoln UK; ^3^ Department of Biological and Experimental Psychology Queen Mary University London London UK

**Keywords:** cross‐modal, face, matching, multimodal, unimodal, voice

## Abstract

Identity verification from both faces and voices can be error‐prone. Previous research has shown that faces and voices signal concordant information and cross‐modal unfamiliar face‐to‐voice matching is possible, albeit often with low accuracy. In the current study, we ask whether performance on a face or voice identity matching task can be improved by using multimodal stimuli which add a second modality (voice or face). We find that overall accuracy is higher for face matching than for voice matching. However, contrary to predictions, presenting one unimodal and one multimodal stimulus within a matching task did not improve face or voice matching compared to presenting two unimodal stimuli. Additionally, we find that presenting two multimodal stimuli does not improve accuracy compared to presenting two unimodal face stimuli. Thus, multimodal information does not improve accuracy. However, intriguingly, we find that cross‐modal face‐voice matching accuracy predicts voice matching accuracy but not face matching accuracy. This suggests cross‐modal information can nonetheless play a role in identity matching, and face and voice information combine to inform matching decisions. We discuss our findings in light of current models of person perception, and consider the implications for identity verification in security and forensic settings.

## BACKGROUND

Verifying the identity of unfamiliar individuals by matching face images or voice samples is important in security and forensic settings such as border control and comparing surveillance recordings in court. Studies using face and voice matching paradigms, where perceivers are presented with two face images or two voice samples and are asked to determine whether the two depict the same person or two different people, report that accuracy is often well above chance. However, at the same time, performance on these tasks is not perfect and can be error‐prone depending on the specific nature of the stimuli and the task (Bruce et al., 1999; Davis & Valentine, 2009; Megreya & Burton, 2006, 2008; Smith et al., 2019; Stevenage & Neil, 2014). Erroneous human judgements about photo ID and speaker discrimination may compromise security and undermine the administration of justice. Although research has explored methods of mitigating the risk of errors by selecting personnel who happen to perform well at these tasks (Davis et al., 2016; Jenkins et al., 2021; Robertson et al., 2016), face matching training (Towler et al., 2017, 2019) and image interactivity (Smith et al., 2021), the existing literature has focused exclusively on unimodal identity decisions and the majority uses only faces. In the current study, we use an identity matching task to investigate whether being able to access the multimodal face and voice information offers a way of optimizing identity verification for both faces and voices.

### Comparing face and voice identity perception

Voice identity perception is overall more error‐prone than face identity perception, which can potentially be explained by voices either contributing less reliable information to identity percepts or by listeners being biased to rely primarily on face information for identity perception (Stevenage & Neil, 2014; Young et al., 2020). The accuracy of identity perception for voices may be less reliable because during interactions voices are only audible while a person is speaking, while faces can be examined for identity information for as long as a person is in view. Further, we likely often prioritize linguistic content and meaning over identity when processing voices (Goggin et al., 1991; Stevenage & Neil, 2014; Young et al., 2020). This may also account for other performance asymmetries reported in the literature, with memory for voices being more subject to interference from same‐modality distractors than faces (Stevenage & Neil, 2014) and evidence for a ‘face overshadowing effect’ whereby the presence of a face is detrimental for voice identity learning (Cook & Wilding, 1997, 2001; Lavan et al., 2023; Stevenage et al., 2011; Tomlin et al., 2017, but see Maguinness et al., 2021; Schall et al., 2013; Sheffert & Olson, 2004; Von Kriegstein et al., 2008; Von Kriegstein & Giraud, 2006; Zäske et al., 2015). In contrast, there have been no reports of the presence of voices interfering with face learning (e.g. Stevenage & Neil, 2014). That said, the differences between face and voice processing should not be overstated.

The literature overwhelmingly points to there being parallels between face and voice perception. For example, effects such as averaging and distinctiveness work in similar ways for both faces and voices (Barsics & Brédart, 2012; Bruckert et al., 2010; Langlois & Roggman, 1990). It is even suggested that the voice might be considered as a kind of ‘auditory face’ (Belin et al., 2004). So while there are some differences in how accurately identity‐related information can be perceived from faces vs. voices, the existing literature and models suggest that face and voice perception can often be integrated and interacting processes (Belin, 2017; Belin et al., 2004; Campanella & Belin, 2007; Maguinness & von Kriegstein, 2017; Young et al., 2020; Yovel & Belin, 2013).

### The potential benefit of multimodal information

According to the auditory face model (Belin et al., 2004), parallel integrated face and voice processing facilitates the exploitation of redundant information (Belin et al., 2011), which is a key reason why multimodal matching might optimize identity verification from faces and voices. Various studies point to the existence of redundant information for auditory and visual cues such as attractiveness, masculinity, femininity, health and emotion (Collins & Missing, 2003; Pourtois & Dhar, 2012; Saxton et al., 2006; Smith et al., 2016a). This kind of concordance has for example been discussed as being adaptive in terms of mate choice, helping to limit incorrect trait assessments (see Moller & Pomiankowski, 1993). It has even been shown that it is possible to match unfamiliar faces to unfamiliar voices (Kamachi et al., 2003; Krauss et al., 2002; Lachs & Pisoni, 2004; Mavica & Barenholtz, 2013; Smith et al., 2016a, 2016b; Stevenage et al., 2017). Although overall performance tends to be low, cross‐modal identity matching is possible, particularly when the facial stimuli are dynamic (articulating but muted) rather than static (Smith et al., 2016a, 2016b, Experiment 3; Huestegge, 2019). In examining whether the visibility of idiosyncratic mouth movements might explain the dynamic advantage (Lander et al., 2001, 2007), Lavan et al. (2021) observed chance level matching for both static and dynamic face matching. These findings partially contradict the extant literature, but the results highlight the fragile nature of face‐voice matching. The discrepancy is likely to be accounted for by stimulus effects, with some people simply looking and sounding more similar than others, and face‐voice matching accuracy varying according to speaker identity (Mavica & Barenholtz, 2013; Smith et al., 2016a, 2016b, 2016c; Stevenage et al., 2017).

Discrepant results across studies and low, seemingly unreliable performance could furthermore be partly explained by face‐voice matching being a crude measure of cross‐modal concordance. Even if faces and voices do provide redundant information for a number of cues (Collins & Missing, 2003; Dhar & Pourtois & Dhar, 2012; Saxton et al., 2006; Smith et al., 2016a), this is not true of all cues (Campanella & Belin, 2007) and accuracy is likely to be frustrated by response biases and stimulus order effects (Lavan et al., 2021; Smith et al., 2016a, 2016b, 2016c; Stevenage et al., 2017). Indeed, Lavan et al. (2022) propose that matching tasks impose a rigid framework that restricts the processing of auditory and visual information. As an alternative, the authors devised and tested a novel audio‐visual sorting task in which participants group video‐only and audio‐only clips into two separate identities. They report that cross‐modal matching was at chance level or below, revealing biases to link the wrong face to the wrong voice. However, the study only involved two identities, such that these effects are likely identity‐specific. A further method of harnessing and measuring cross‐modal concordance is to adapt and expand traditional unimodal matching tasks by including multimodal information. In turn, this may help people to navigate unpredictable variability which can make unimodal matching challenging.

### The role of stimulus variability

Research in face and voice perception has historically treated stimulus variability as a source of noise. Studies have as a result tried to control all but one aspect of the stimuli, that is, the variable of interest, such as expression or head angle for faces, or speech content or speaking style for voices. The resulting tightly controlled stimulus sets, however, do not reflect the way we encounter people in real life and it has more recently been argued that controlling away variability may in fact also remove the very cues we use to recognize people in the real world (Burton, 2013). Current research in both face and voice perception tends to use ‘ambient’ stimuli which capture the within‐person variability encountered in the real world (e.g. Lavan et al., 2019; Ritchie & Burton, 2017).

Variability generally presents a challenge to accurate face and voice identity perception alike. For example, facial appearance can vary according to lighting conditions or pose, while the sound of a voice can vary according to the intended audience or environment acoustics. When someone is unfamiliar it can be difficult to predict how, and to what extent, the appearance of their face or the sound of their voice might change in different circumstances. Indeed, within‐person variability appears to be idiosyncratic and difficult to predict (Burton et al., 2016; Lavan et al., 2019; Lee et al., 2019; Young et al., 2020). To achieve accurate identity perception, variation from a wide range of sources must therefore be tolerated, as the same person might look or sound quite different across instances (Burton, 2013; Lavan et al., 2019). In some circumstances, between‐person variability can also pose challenges to accurate identity perception, with different people exhibiting coincidental facial or vocal similarity (Fleming et al., 2014; Valentine et al., 2016). Both face and voice identity matching decisions are, therefore, clouded by the difficulty of deciding whether differences between faces or voices should be attributed to within‐person or between‐person variability. It thus follows that the accuracy of face and voice identity perception could be improved by perceivers being able to correctly perceive within‐ and between‐person variability as such. To this end, having a concordant other‐modality stimulus available as a reference point in multimodal matching may be helpful.

In the current study, we examine whether and how having access to multimodal identity information may enhance identity matching performance. Across three experiments, we test the potential effects of the presence of multimodal information on identity matching for faces and voices, while also relating our findings back to how the perception of concordant identity‐related information might contribute to these matching decisions.

### EXPERIMENT 1

In Experiment 1, we investigated the basic question of whether having access to identity‐related information from both the face and the voice can enhance accuracy for identity matching compared to unimodal identity matching, where information from only one modality is present (i.e. the face or the voice). To do this, we asked participants to complete an identity matching task with either two unimodal stimuli (i.e. determining whether two silent videos of faces or two recordings of voices showed the same person or two different people) or with multimodal information being present (i.e. determining whether a silent video/voice recording and an audiovisual stimulus including both face and voice information show the same person or not).

In Experiment 1 we also compare performance across the same identity and different identity trials. Matching accuracy often differs across trial types (e.g. Ritchie et al., 2021; Smith et al., 2021), with participant‐wise accuracy on same vs. different identity trials being uncorrelated (Megreya & Burton, 2007). For both faces and voices, accuracy across trial types is likely to vary according to the specific task, but the direction is difficult to predict. For example, accuracy is higher on different identity voice matching trials when background noise is present compared to when it is absent (Smith et al., 2019), and while errors on different identity face matching trials tend to be more common than same identity errors (Davis & Valentine, 2009; Kemp et al., 1997), this is not the case when matching interactive facial images (Smith et al., 2021).

If faces and voices provide at least some perceptually salient redundant information about a person's identity (or other perceptually meaningful characteristics), we predict that having access to multimodal information should be associated with more accurate performance than when only unimodal information is present. Specifically, we expect that the addition of the ‘other’ modality in the audiovisual stimuli may help to triangulate the matching decision, making it easier to compartmentalize within‐person and between‐person variability by providing an additional point of reference. That is, in a face matching task, where a voice stimulus is present, for example, not only can participants' decisions draw on consideration of unimodal similarity/dissimilarity but also implicit consideration of whether the voice veridically belongs to the same identity face (audiovisual stimulus) is a likely match to the unimodal face. If face and voice cues are either not sufficiently concordant to indicate a common source or any concordances are not perceptually salient, the ‘other’ modality information will not improve matching accuracy compared to unimodal matching.

Based on previous findings, we also expect face‐face matching to be more accurate than voice‐voice matching (Stevenage & Neil, 2014). In line with this prediction, we do not expect multimodal information to have symmetrical effects on face and voice matching. As face perception is more reliable than voice perception, audiovisual stimuli may support identity verification from voices to a greater extent than faces, because the additional information is more diagnostic. We are not aware of studies comparing unfamiliar face and voice matching across different tasks, but have no reason to predict that trial type will interact with modality.

## METHODS

### Participants

199 participants (106 male) were recruited via Prolific.co. All participants were aged between 20 and 76 years (*M* = 38.83, *SD* = 12.16), had no self‐reported hearing impairments, normal or corrected‐to‐normal vision, spoke English as their first language and had grown up and currently lived in England. All participants had a minimum approval rate of 90% on Prolific.co, having previously completed a minimum of 5 online studies. Participants were compensated for their time at a rate of £10.50 per hour of planned participation.

All participants passed the attention checks (see below), such that no exclusions were made. All experiments were approved by the research ethics committee at Nottingham Trent University (Application ID: 1610103).

Fifty participants (29 male) took part in the voice matching task (age: *M* = 38.80 years, *SD* = 12.56 years), 49 participants (29 male) took part in the face matching task (age: *M* = 37.49 years, *SD* = 11.37 years), 51 participants (30 male) took part in the voice‐facevoice matching task (age: *M* = 37.84 years, *SD* = 11.64 years) and 49 participants (18 male) took part in the face‐facevoice matching task (age: *M* = 41.24 years, *SD* = 13.02 years).

### Materials

#### Stimuli

The stimuli were videos of 48 identities talking to the camera. All videos were sourced from YouTube. We used white local celebrities from Canada and Australia specifically chosen to be unfamiliar in the UK (12 Australian females, 12 Australian males, 12 Canadian females, 12 Canadian males) and extracted two videos per identity. The celebrities were all aged between approximately 25–50 years old. The videos showed the celebrities in a broadly frontal pose talking to the camera as themselves (i.e. not acting or reading from a script). No other voices or background music were audible. Videos were cropped using an online video cutter (https://online‐video‐cutter.com/) to show just the head and shoulders (4:3 aspect ratio) during a 2–3 s meaningful utterance, for example, “I was about seventeen”. All video clips were resized to a height of 300 pixels. All further editing was done using Premier Pro with the audiotracks being normalized for peak intensity across all clips.

We then created audio‐only versions (by removing the video) and video‐only versions (by muting the audio track) from these audiovisual stimuli to build different types of face and/or voice matching tasks. All audio‐only files were saved as MP3 files, and all files including visual information were saved as MP4 files.

### Procedure

The task was completed in the Gorilla Online Experiment Builder (www.gorilla.sc; Anwyl‐Irvine et al., 2020). After giving informed consent, participants in all experiments completed a check to ensure that audio playback was working appropriately and that their volume was set to a comfortable level. They listened to a sound file of a spoken word and were required to correctly type the word before continuing.

**FIGURE 1:**
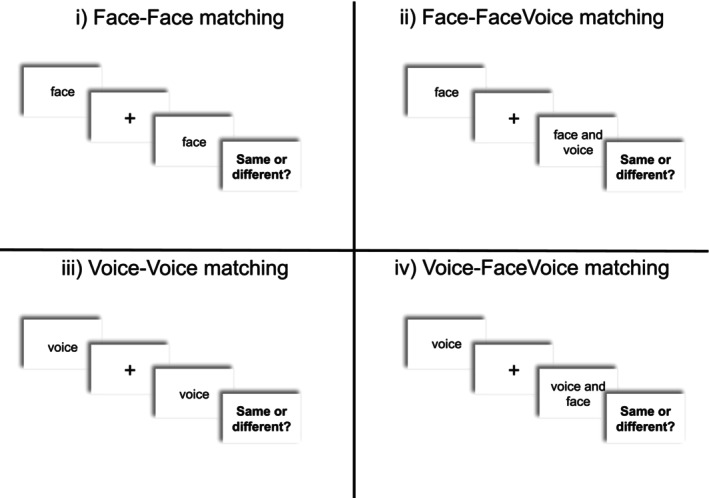
Illustration of the trial procedure in each of the 4 matching tasks in Experiment 1.

Following this, participants completed one of four different tasks (see Figure 1): (i) face‐face matching (ii) face‐facevoice matching (iii) voice‐voice matching or (iv) voice‐facevoice matching.

For face‐face matching, participants were sequentially^1^ shown two video‐only clips of faces (i.e. there was no voice information present), while for face‐facevoice matching participants were shown one video‐only clip and one multimodal clip, with the order of the unimodal and multimodal stimulus counterbalanced within participants. Similarly, for voice‐voice matching participants were sequentially presented with two audio‐only clips of voices (i.e. there was no face information present), while for voice‐facevoice matching participants were shown one audio‐only clip and one multimodal clip. The stimulus interval was 500 ms. For all tasks, participants were asked to indicate whether they thought that the two stimuli featured the same person or two different people. They were also informed that ‘The people in the videos are saying different things. What they are saying will not help you to complete the task’. Participants completed a practice trial before progressing to the main experiment. Stimuli from the practice trials were not reused in the main experiment. In the main experiment, the order of stimuli in each trial was counterbalanced across participants.

The matching task included 48 matching identity trials, including trials from 24 pairs of Australian identities (12 female) and 24 trials of Canadian identities (12 female). Identities in each sub‐group of 12 (sex, country of origin) were equally assigned to the same identity or different identity trials. In total, there were 24 same identity trials and 24 different identity trials. For identities in ‘same’ trials, we used two different videos of them taken on two separate occasions showing variability in lighting, facial expression, hairstyle etc. Different identity pairings were made on the basis that both identities fit the same verbal description (e.g. young woman, dark hair) and were chosen by the experimenters. Each participant saw each identity once. We included an additional 6 trials as attention checks. In these trials, participants completing the face‐face and face‐facevoice matching tasks were instructed to ‘Please select “same person”/“different people”’ via a text prompt on the screen. Participants completing the voice‐voice and voice‐facevoice matching tasks received the same instruction via a sound file of a voice reading the instructions. All trials were fully randomized.

### Data analysis

We analysed trial‐wise accuracy data (0 = incorrect, 1 = correct) using generalized linear mixed models (GLMM) using the R package lme4 (Bates et al., 2015). A multilevel approach is preferable to using ANOVA because it accounts for the random variability associated with stimuli and participant performance. Variability at the stimulus level in face‐voice matching means that aggregating data involves making false assumptions about patterns of performance across individual trials (Smith et al., 2016b). Statistical significance of main effects and interactions (where present) were taken from the output of the *glmer* function, odds ratios and associated confidence intervals were calculated via the *tab_model* function in the R package *sjplot*. Model structures for each mode and reference levels for each factor are described within the results section. Post‐hoc simple main effects were computed using the R package emmeans, where alpha was in all cases Bonferroni‐corrected for multiple comparisons (see Results section).

## RESULTS

### Matching task and other‐stimulus modality

We first ran a GLMM to assess whether having access to multimodal information during face matching and voice matching tasks improves accuracy. In this model, we included the matching task (face (reference level) vs. voice) and other‐stimulus modality (multimodal facevoice (reference level) vs. unimodal face/unimodal voice) as fixed effects, including the main effects and interaction. The random effects structure included the participant, the first stimulus and the second stimulus of each matching trial, alongside the country of origin for each identity sampled (Australia vs. Canada). We included the country of origin because we could not rule out the possibility that these sets of stimuli differed from each other in a systematic way and that there was variability in matching performance associated with these differences. Stimulus gender, person identity and presentation order of the stimuli all resulted in singular fits, such that they were not included in the model.

**FIGURE 2:**
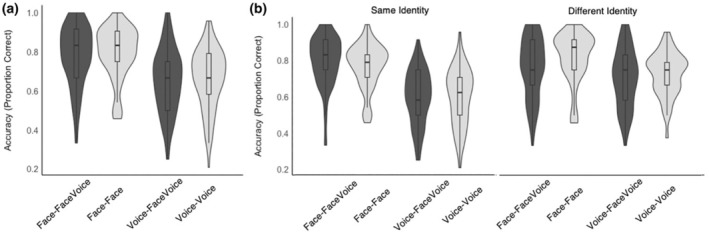
(a) Accuracy for face and voice identity matching, when only unimodal (for face‐face and voice‐voice matching) or multimodal information (for face‐facevoice and voice‐facevoice matching) is available. (b) Accuracy for face and voice identity matching split by “same identity” and “different identity” trials, when only unimodal or multimodal information is available.

Data are plotted in Figure 2a. There was no two‐way interaction between the matching task and other‐stimulus modality (*b* < 0.01, *z* = 0.04, *p* = 0.966, *OR* = 1.01, CI 0.71–1.42) and no main effect of other‐stimulus modality (*b* = 0.04, *z* = 0.35, *p* = 0.729, *OR* = 1.04, CI 0.82–1.32). There was, however, a main effect of matching task (*b* = −0.69, *z* = −3.68, *p* < 0.001, *OR* = 0.50, CI 0.35–0.72), with accuracy for face matching (face‐face: *M* = 0.79, *SD* = 0.13; face‐facevoice: *M* = 0.78, *SD* = 0.15) being higher than for voice matching (voice‐voice: *M* = 0.66, *SD* = 0.15; voice‐facevoice: *M* = 0.65, *SD* = 0.16). Contrary to our predictions, this analysis shows that accuracy did not increase for face or voice matching when participants had access to multimodal information for one of the stimuli in a matching trial.

### Matching task, other‐stimulus modality and trial type

To test whether there were any effects of other‐stimulus modality for the different trial types (same vs. different identity), we, therefore, ran an additional GLMM: This model was the same as the one reported above, however, now including trial type (different (reference level) vs. same identity within a stimulus pair) in addition to the matching task (face vs. voice), other‐stimulus modality (multimodal facevoice (reference level) vs. unimodal face/unimodal voice) as fixed effects, with all interactions. The random effects structure was identical.

Data are plotted in Figure 2b. There was a three‐way interaction between the factors (*b* = 0.81, *z* = 2.80, *p* = 0.005, *OR* = 2.25, CI 1.28–3.97). There was also a two‐way interaction between other‐stimulus modality and trial type (*b* = −0.67, *z* = −3.41, *p* = 0.001, *OR* = 0.51, CI 0.35–0.75) and matching task and trial type (*b* = −1.11, *z* = −3.24, *p* = 0.001, *OR* = 0.33, CIs 0.17–0.65). There was no two‐way interaction between matching task and other‐stimulus modality (*b* = −0.41, *z* = −1.77, *p* = 0.078, *OR* = 0.67, CI 0.42–1.05). In the presence of interactions with all of the factors, main effects are of limited interpretability and thus not reported here. The significant interactions are explored further below via looking at simple main effects of interest between the levels of these three factors.

Trial type (same vs. different) significantly interacted with other‐stimulus modality: When running comparisons between the conditions of these two factors, accuracy was significantly higher for unimodal (i.e. face‐face and voice‐voice) ‘different’ trials than for unimodal ‘same’ trials (*z* = 2.84, *p* = .005), which is driving the interaction. This represents more conservative responding patterns for the face‐face matching task, where participants less readily accepted two stimuli as representing the same identity than they did in the face‐facevoice condition. Please see the Data S1 for a supplementary signal detection analysis using Criterion C and D Prime, which confirm the more conservative response pattern for face‐face matching. As can be seen in Figure 2b, this interaction seems to be mainly driven by the face‐face matching conditions. All other comparisons between conditions were not significantly different from one another (*z* < 2.03, *p* > .042; *α* = .008; corrected for 6 comparisons). The trial type also interacted with the matching task. Simple main effects showed, that the difference in accuracy for the same identity trials between face vs. voice matching tasks was larger (*z* = 4.68, *p* < .001) than the difference in accuracy for different identity trials (*z* = 1.54, *p* = .123).

To address our main research question, which was to examine the effect of multimodal information at the trial‐type level, we directly examine whether having access to multimodal information affects accuracy for face or voice matching in same or different identity trials. As in the matching task and other‐stimulus modality analysis reported above, simple main effects (*α* = .0125, Bonferroni‐corrected for 4 comparisons) between ‘unimodal’ face‐face and voice‐voice and ‘multimodal’ face‐facevoice and voice‐facevoice conditions suggest that there is no significant increase in voice matching accuracy for either same identity trials (*z* = 0.44, *p* = .660) or different identity trials (*z* = 0.08, *p* = .936). For face matching simple main effects are also not significant for both the same identity (*z* = 2.30, uncorrected *p* = .021) and different identity trials (*z* = −1.70, uncorrected *p* = .087) after correction for multiple comparisons.

### Discussion Experiment 1

Contrary to our predictions, there is no evidence in our data to suggest that having access to information from both the face and the voice as part of a matching task increases the accuracy of identity matching. However, multimodal information had a small effect on how readily participants accepted two stimuli as representing the same identity. These findings therefore suggest that there is either no perceptually meaningful, concordant information about a person's identity provided by faces and voices or–in the case where shared or concordant identity‐related information exists–that this information is not used by participants to improve matching accuracy.

### EXPERIMENT 2

Experiment 1 showed no clear evidence that being able to access identity‐related information from another modality as part of one of the stimuli within a matching trial can improve matching accuracy. In Experiment 2, we asked whether having access to multimodal information for *both*, compared to neither, stimuli in a matching trial would give people more information and improve accuracy. This was intended as a formal test to examine the usefulness of multimodal information during matching tasks. We predicted that consistent access to multimodal information within a matching trial would improve the accuracy of identity matching compared to only having access to information from the face due to more information being available to perceivers. In line with our aim of examining whether access to multimodal identity information enhances identity matching, we focussed on comparing the new multimodal matching data to the face‐face matching data only, because Experiment 1 shows that (1) matching accuracy for faces is higher than for voices and (2) accuracy is similar for the two face matching tasks, albeit numerically higher for the face‐face matching condition (0.66 vs. 0.65).

## METHODS

Apart from the following exceptions, the methods were the same as Experiment 1.

### Participants

Fifty‐one participants (24 male) were recruited via Prolific.co. All participants were aged between 20 and 72 years (*M* = 39.55, *SD* = 14.77). All participants passed the attention checks, such that no exclusions were made. Participants were sampled with the same criteria and compensated at the same rate as Experiment 1.

### Procedure

Participants completed a fully multimodal matching task. Unlike the face‐facevoice matching and voice‐facevoice matching tasks used in Experiment 1, participants were sequentially shown two multimodal clips (facevoice‐facevoice). Experiment 2 used the same trial structure as Experiment 1.

Three of the 6 attention check trials (i.e. ‘Please select “same person”/“different people”’) were presented as visual text prompts on the screen and the other 3 were presented as auditory instructions, to make sure participants paid attention to both modalities.

### Results

In this analysis, we compare the face‐face matching condition from Experiment 1 to the new facevoice‐facevoice data. To test whether having access to multimodal information for both stimuli in the matching task significantly increases accuracy compared to face‐face matching, we ran a GLMM, including the matching task (face‐face (reference level) vs. facevoice‐facevoice) and the trial type (different (reference level) vs. same) as fixed effects, including the two main effects and the interaction. The random effects structure was the same as in Experiment 1.

**FIGURE 3:**
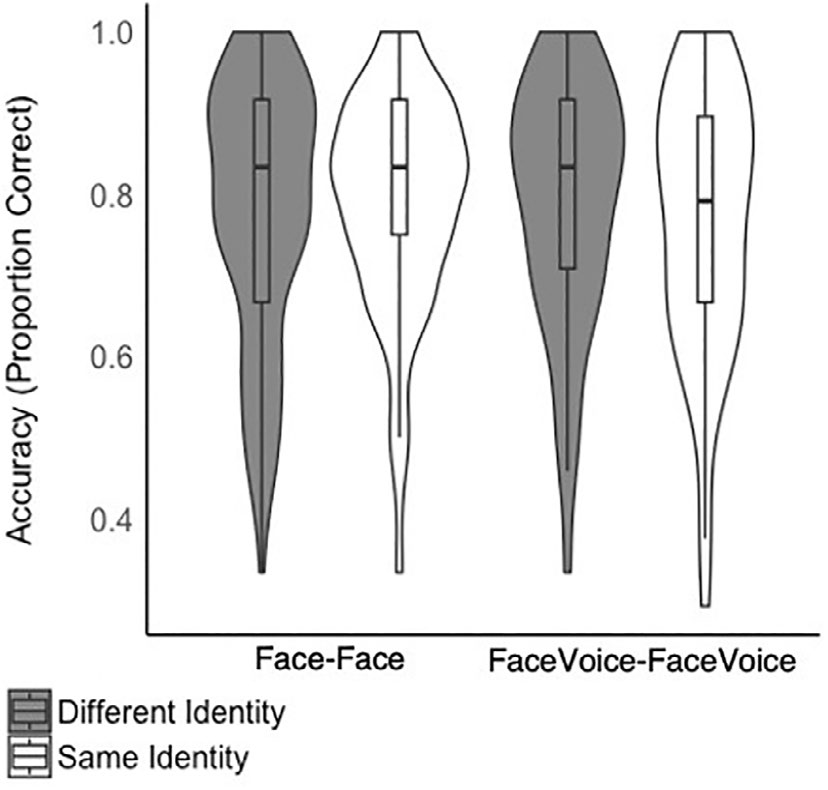
Accuracy for face‐face and facevoice‐facevoice identity matching, split by “same identity” and “different identity” trials.

Data are plotted in Figure 3. There was no two‐way interaction between the factors (*b* = 0.16, *z* = 0.56, *p* = .576, *OR* = 1.18, CI 0.67–2.08), neither were there main effects of trial type (*b* = −0.32, *z* = −1.53, *p* = .126, *OR* = 0.73, CI 0.48–1.09) or matching task (*b* = −.21, *z* = −0.88, *p* = .379, *OR* = 0.81, CI 0.52–1.29). This result is underlined by the means for facevoice‐facevoice matching (*M* = 0.77, *SD* = 0.16) being very similar to the means for face‐face matching (*M* = 0.79, *SD* = 0.13). Having consistent access to multimodal identity information—as opposed to having only access to information from the face—does not significantly increase matching accuracy.

### Discussion Experiment 2

Again, contrary to our predictions, having consistent access to identity information from both auditory and visual modalities during an identity matching task had no significant effect on accuracy compared to the face‐face matching task. These results, therefore, suggest that despite including some information about a person's identity, vocal identity information is not used by perceivers to enhance their performance on a matching task because they rely overwhelmingly on face identity information.

### EXPERIMENT 3

Previous research has shown that cross‐modal identity matching (i.e. determining whether a voice and a face belong to the same identity) is under certain circumstances possible. That is, matching accuracy has been found to be significantly above the chance level, albeit with low accuracy. However, these studies also often highlight substantial item‐based effects, such that some face‐voice combinations are, for example, consistently and accurately identified as coming from the same person (Mavica & Barenholtz, 2013; Smith et al., 2016a, 2016b, 2016c; Stevenage et al., 2017). Others are, however, consistently mismatched, that is, participants think a face and voice belong to the same person when they are in reality sampled from two different people (Lavan et al., 2022). In Experiment 3, we build on these observations, examining: (1) how well participants can on average cross‐modally match faces to voices in our stimulus set by comparing performance to chance level and (2) whether item effects can shed more light on the results from Experiments 1 and 2. Specifically, we run GLMMs to address whether the accuracy of cross‐modal face‐voice matching predicts the accuracy of face‐facevoice and voice‐facevoice matching. If it does, this would indicate that cross‐modal information is to some degree used when making voice and face matching decisions, despite the observed lack of improvement in accuracy observed in Experiments 1 and 2. As before, we predicted that possible relationships may be asymmetrical for face and voice matching, such that the effect of cross‐modal information was predicted to be bigger for voice matching than for face matching. We reasoned that the relative unreliability of vocal identity information over facial information (Young et al., 2020) might encourage perceivers to rely on face information when available for one stimulus in a voice matching task.

## METHODS

Apart from the following exceptions, the methods were the same as Experiment 1.

### Participants

Fifty‐two participants (29 male) were recruited via Prolific.co. All participants were aged between 22 and 70 (*M* = 38.98, *SD* = 12.06). All participants passed the attention checks, such that no exclusions were made. Participants were sampled with the same criteria and compensated at the same rate as Experiments 1 and 2.

### Procedure

Participants completed a cross‐modal face‐voice matching task. They were sequentially shown a video‐only (i.e. muted voice) clip and an audio‐only clip, with the order of the stimulus modalities counterbalanced within participants. Experiment 3 used the same trial structure as Experiment 1, with the order of stimuli in each trial counterbalanced across participants.

As in Experiment 2, to make sure participants paid attention to both modalities 3 of the 6 attention check trials (i.e. ‘Please select “same person”/“different people”’) were presented as visual text prompts on the screen and the other 3 were presented as auditory instructions.

### Results

#### Comparison to chance‐level

**FIGURE 4:**
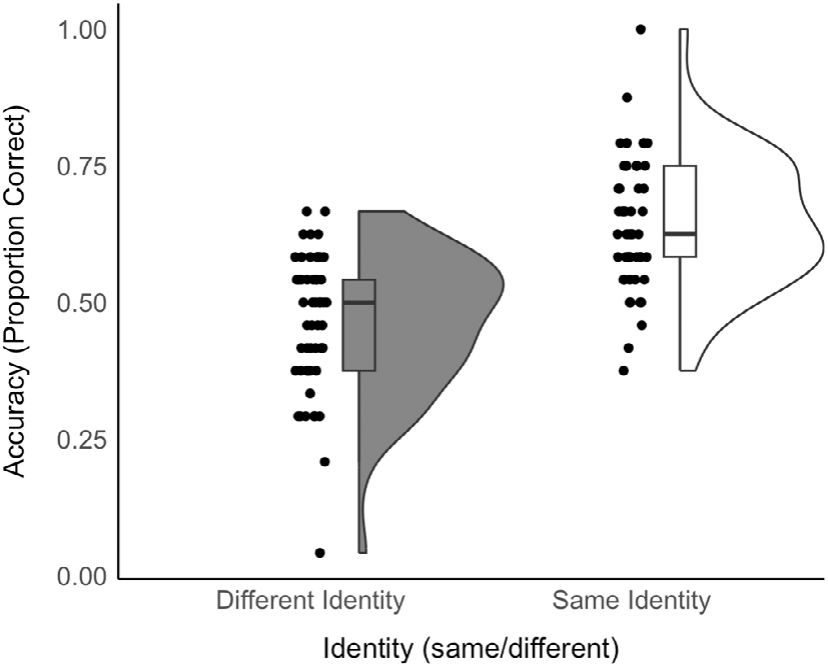
Raincloud plots show the accuracy of face‐voice matching, split by trial type. Black dots show the accuracy of individual stimulus pairs.

The mean accuracy for face‐voice matching was 0.55 (*SD* = 0.15); with accuracy for the same identity trials being 0.46 (*SD* = 0.12) and accuracy for different identity trials being 0.65 (*SD* = 0.13, see Figure 4). We first established whether the accuracy for cross‐modal face‐voice matching was above chance (0.5). For this purpose, we ran a GLMM including identity as a fixed effect with no intercept. We obtained 95% confidence intervals (CIs) by simulating the posterior distributions of the cell means in *R* (*arm* package; Gelman & Su, 2013). The confidence intervals overlapped with 0.5 for different identity trials (95% CI [0.34; 0.57]), showing that accuracy was at chance. Confidence intervals did not overlap with 50% for the same identity trials (95% CI [.57; .77]), showing that accuracy was above chance. This difference in accuracy between trial types (same vs. different) was also significant (*b* = 0.75, *z* = 3.15, *p* = .001, OR = 2.12, CI 1.33–3.39).

### Cross‐modal matching: the relationship between face and voice matching

**FIGURE 5:**
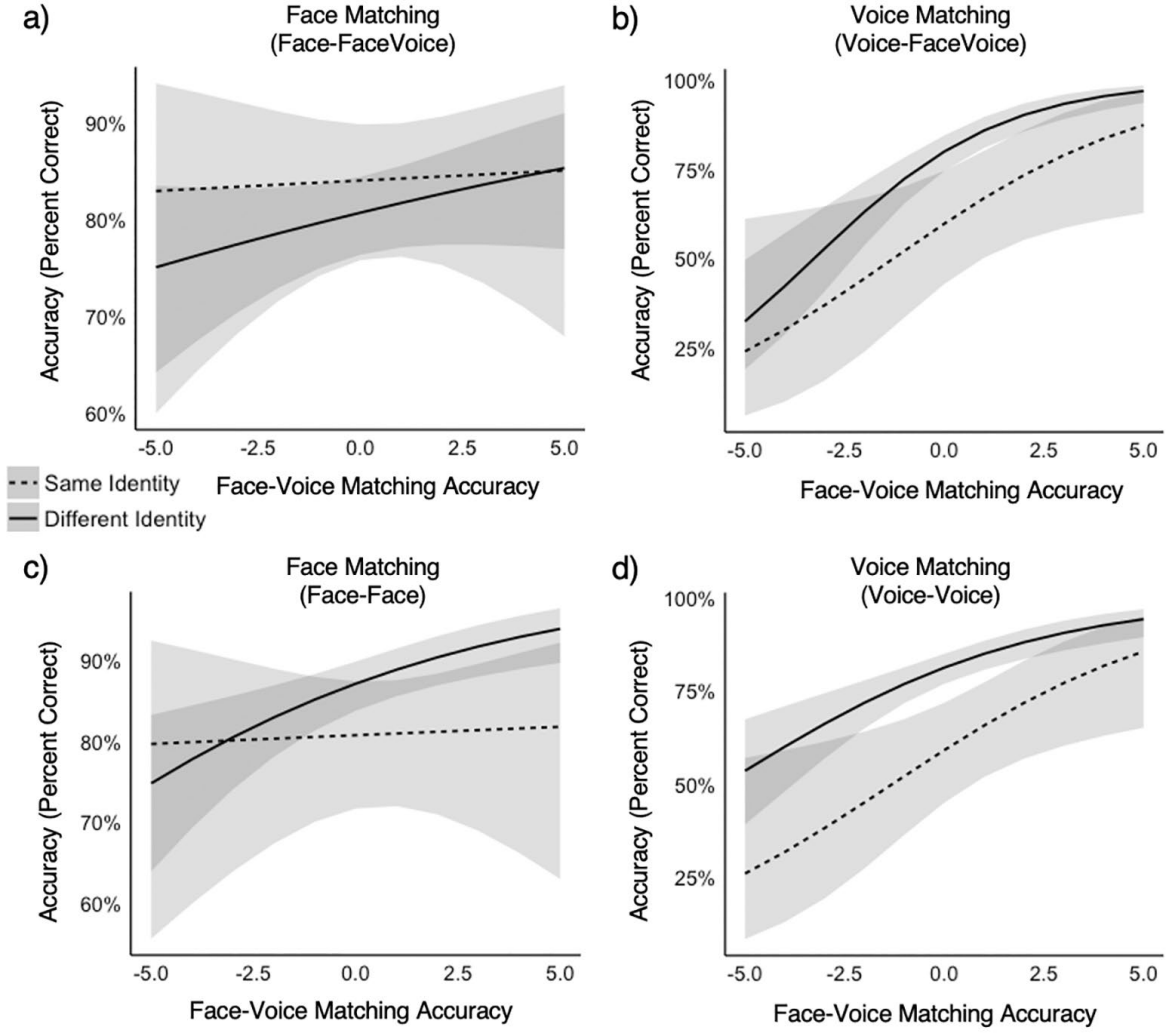
Predicted relationships between cross‐modal matching accuracy and (a) face‐facevoice matching accuracy, (b) voice‐facevoice matching accuracy, (c) face‐face matching accuracy and (d) voice–voice matching accuracy. Grey bands around the trendlines show 95% confidence intervals. Plots show the centred and re‐scaled face‐voice matching accuracy data.

We examined whether perceived shared cross‐modal identity information (data from Experiment 3) can predict accuracy for face or voice matching when multimodal information is present in one of the stimuli within a matching trial (data from Experiment 1). To do this, we ran two GLMMs (for face matching and voice matching separately) with trial type and the cross‐modal face‐voice matching accuracy as fixed effects. Cross‐modal face‐voice matching accuracy was centred and re‐scaled (to deciles). The trial type (same vs. different) was coded via deviation coding (−0.5; 0.5) in line with centring the fixed effect of face‐voice matching accuracy. Random effects were the same as for all other analyses, with the nationality of the identity not being modelled as it led to singular fits. For face matching, there was as predicted no interaction between trial type and face‐voice matching accuracy (*b* = −0.05, *z* = −0.52, *p* = .602, *OR* = 0.95, CI 0.79–1.15), nor was there a main effect of trial type (*b* = 0.23, *z* = 1.00, *p* = .318, *OR* = 1.26, CI 0.80–1.97) or face‐voice matching accuracy (*b* = 0.04, *z* = 0.85, *p* = .396, *OR* = 1.04, CI 0.95–1.14), (see Figure 5a). For voice matching, we found no interaction between trial type and face‐voice matching accuracy (*b* = −0.12, *z* = −0.81, *p* = .417, *OR* = 0.89, CI 0.67–1.18). There was an effect of trial type (*b* = −0.99, *z* = −3.11, *p* = .002, *OR* = 0.37, CI 0.29–0.69) and a main effect of face‐voice matching accuracy (*b* = 0.37, *z* = 5.18, *p* < .001, *OR* = 1.45, CI 1.26–1.66; see Figure 5b). As predicted, this suggests that, for voice matching participants make matching decisions in a way that can be linked back to how well the faces and voices appear to go together as indexed by cross‐modal identity matching accuracy. This was, however, not the case for faces, where face matching accuracy could not be linked to cross‐modal matching accuracy.

In case these results could be explained by something other than the multimodal nature of the stimuli, as a ‘control’ analysis we ran the same GLMMs again, however, now considering the data where only one modality was available through the matching trials, again for face and voice matching separately. Given that no information from the other modality was present, no relationships between cross‐modal face‐voice matching accuracy and face‐face or voice‐voice matching should be apparent. For face matching, there was again no interaction between trial type and face‐voice matching accuracy (*b* = −0.15, *z* = −1.57, *p* = 0.117, *OR* = 0.86, CI 0.71–1.04) and no main effect of face‐voice matching accuracy (*b* = 0.09, *z* = 1.85, *p* = 0.064, *OR* = 1.09, CI 0.99–1.20, see Figure 5c). There was a main effect of trial type (*b* = −0.48, *z* = −2.1, *p* = 0.036, *OR* = 0.62, CI 0.40–0.97).

For voice matching, we found no interaction between trial type and face‐voice matching accuracy (*b* = 0.02, *z* = 0.11, *p* = .876, *OR* = 1.02, CI 0.81–1.27). There were, however, again main effects of trial type (*b* = −1.09, *z* = −4.10, *p* < .001, *OR* = 0.33, CI 0.20–0.56) and face‐voice matching accuracy (*b* = 0.27, *z* = 4.77, *p* < .001, *OR* = 1.31, CI 1.17–1.47, see Figure 5d). These findings thus surprisingly replicated the findings from the analysis of the face‐facevoice and voice‐facevoice matching data.

### Discussion Experiment 3

As in previous research, we find that cross‐modal face‐voice matching is possible under certain circumstances. Here, we found that while accuracy was not above chance for cross‐modal identity judgements for matching trials that included two different identities, accuracy was above chance when matching trials included the same identity. Furthermore, our data support previous studies showing that some people look and sound more similar than others (Mavica & Barenholtz, 2013; Smith et al., 2016a, 2016b, 2016c; Stevenage et al., 2017), with levels of matching accuracy varying widely across face‐voice pairings.

We find that cross‐modal face‐voice matching accuracy predicts both voice‐facevoice and voice‐voice matching accuracy—but does not predict face matching accuracy in any trial format. It is plausible that voice matching accuracy is contingent on whether or not the face and voice look and sound like they belong to the same person in the multimodal condition. While the result for voice‐facevoice matching aligns with our predictions, the voice‐voice matching results are surprising as even when no face information was available, underlying face‐voice concordance predicts performance. When considering the comparative dominance and reliability of faces over voices in identity processing (Young et al., 2020), it perhaps makes sense that even when facial information is unavailable, people might default to attempting to reconcile vocal identities by appealing to a ‘visual’ explanation. One explanation of our data, therefore, could be that when people listen to a voice they generate a visual expectation of what they think that person might look like, with accurate expectations supporting accurate voice matching, and inaccurate expectations undermining accuracy. Imaging studies may speak to such mechanisms: these studies reveal crosstalk between face‐selective and voice‐selective areas (Blank et al., 2011; Von Kriegstein et al., 2005; Von Kriegstein & Giraud, 2006). While the imaging literature focuses on familiar person perception, we could speculate that to some extent, similar processes might operate in unfamiliar person perception. While people might call to mind an image of a face when listening to a familiar person speak (Kriegstein et al., 2005), they might base an expectation of visual appearance on broad demographics or stereotypes while listening to an unfamiliar person speak. For example, a voice that sounds very masculine may lead participants to only accept voices/faces that are perceived to look very masculine as an identity match. Such an approach might not always be reliable: perceived information about attractiveness, masculinity/femininity, health and height (Collins & Missing, 2003; Pourtois & Dhar, 2012; Saxton et al., 2006; Smith et al., 2016a) is shared across faces and voices, which could form a functional basis for generating visual expectations during voice matching. However, while some cues are concordant, other cues are likely to be discordant (Campanella & Belin, 2007). This could in turn explain error‐prone face‐voice matching. Nonetheless, the existence of shared information across some dimensions might bias people to overgeneralize to an expectation of shared information across other dimensions, such that considering potential visual appearance when listening to a voice seems reasonable, and, therefore, contributes to (subjective) identity perception.

## DISCUSSION

In the current study, we asked whether having access to multimodal information can increase accuracy for face and voice identity matching. Contrary to our predictions, the availability of multimodal face‐voice information in a matching task does not optimize unfamiliar identity verification. However, we present evidence that the perception of cross‐modal face‐voice concordance nonetheless has an effect on voice matching performance but not face matching performance.

### Multimodal information does not improve matching accuracy

Experiment 1 shows that while face‐face matching is more accurate than voice‐voice matching, accuracy did not increase for either type of matching when one of the stimuli in a trial was multimodal. The results of Experiment 2 show that even when multimodal information is available for both stimuli in a matching task, performance does not differ from face‐face matching. The presence of other‐modality information, therefore, appears to provide no overall benefit to identity matching tasks over and above the information provided by faces alone. Overall, these findings first underline the unreliability of voice identity perception in comparison to face identity perception (Stevenage & Neil, 2014; Young et al., 2020) and further highlight that participants are unable to use potentially concordant identity‐related information to enhance their matching performance. The only changes in performance between unimodal (face‐face and voice‐voice) and multimodal matching (face‐facevoice and voice‐facevoice) were found in Experiment 1, where participants responded more conservatively when only unimodal information was available. Thus, although concordant face‐voice information does not enhance accuracy overall, it may contribute to multimodal matching decisions, but only to the extent that it shifts the source of error from different identities to the same identity responses.

The stimulus set used in this study was intended to replicate as closely as possible how people perform person perception in the real world. Using ambient stimuli sampling realistic within and between‐person variability, we speculated that having a concordant other‐modality stimulus available as a reference point may support the accurate categorization of different types of variability in comparison to a situation where only unimodal information was available. There was no direct evidence in our study that this was the case.

### The perception of concordant cross‐modal information

Interestingly, Experiment 3 suggests that participants may make use of *perceived* concordant information. As reported previously, cross‐modal face‐voice matching was error‐prone in our study (see also Lavan et al., 2021; Smith et al., 2016a, 2016b; Stevenage et al., 2017). Specifically, participants were not able to tell faces and voices apart above chance level, but they were able to tell them together with poor, but above‐chance accuracy. This pattern of liberal responding has been observed in other face‐voice matching studies (Smith et al., 2016a; Stevenage et al., 2017), and may help to explain why the source of error shifts from different identity to same identity responses in face‐facevoice and voice‐facevoice matching (Experiment 1).

However, intriguingly, cross‐modal face‐voice matching accuracy (Experiment 3) predicted both voice‐facevoice and voice‐voice matching accuracy (Experiment 1). This finding is unique to voices; as no similar relationship was observed for face matching. This finding may shed light on cognitive processes implicated in voice perception. Voice matching accuracy is contingent on whether the face and voice look and sound like they belong to the same person in the multimodal voice‐facevoice condition. We speculate that even in the voice‐voice matching condition, when listening to a voice participants may generate a broad visual expectation of appearance based on general demographic information, upon which they base their identity matching decisions within or across modality. If this expectation aligns with reality, face‐voice matching is possible and both voice‐facevoice and voice‐voice matching are supported. However, if the expectation does not align with reality, perhaps because the face and voice exhibit discordant information, cross‐modal matching is more difficult and so neither multimodal or unimodal matching are supported. We note that different sets of participants completed the unimodal, multimodal and cross‐modal matching tasks in Experiments 1 and 3. However, previous studies reveal that people tend to agree about whether faces and voices look and sound similar. While there is variability at the stimulus level, individual differences in perception are negligible (Smith et al., 2016a, 2016b, 2016c).

More broadly, the results from Experiment 3 may also help to account for some of the errors in unfamiliar voice matching: Whereas in unfamiliar face matching people are able to utilize maximally reliable visual identity information, in unfamiliar voice matching they are influenced by inherently *unreliable* information which is based on the expectation, rather than a ground truth, of face‐voice concordance. The wide variation in matching accuracy across face‐voice pairings may reveal why the results of Experiment 1 show that face information does not support voice matching. Face‐voice pairings that veridically look and sound similar, and which *do* support accurate matching are cancelled out by the effect of face‐voice pairings which do not.

## Conclusion

Taken together, the results of these 3 experiments shed light on how face and voice information combine during identity matching. In face matching, voice information appears to be of little or no consequence, whether multimodal information is present or absent. In voice matching, face information and the perception of cross‐modal concordance influence both multimodal and unimodal matching decisions, regardless of whether the information is veridical or not. The findings are broadly in line with other evidence suggesting that facial information is dominant and overall more informative for identity perception (e.g. Barsics, 2014). While information from faces and voices thus may hold concordant information about a person's identity in some cases, overall this information does not increase accuracy for either face or voice matching.

From an applied perspective, our study therefore shows that having access to multimodal identity information would be unlikely to substantially improve identity verification. Indeed, the results actually warn against including multimodal information because of the associated bias to respond ‘same’. In contexts such as border control, for example, erroneous ‘same’ responses might undermine security and prevent the detection of identity fraud. That said, our results do offer some potential for improving voice identity verification. The presence of multimodal information does not mitigate the risk of unimodal matching errors unless the voice in question sounds like it belongs to its corresponding face. When the ground truth is unknown, the ability to predict voice‐voice matching performance from cross‐modal matching could be important. In the future, studies might develop this line of research such that it becomes possible to improve unfamiliar voice matching performance by encouraging listeners to resist the generation of inherently unreliable visual expectations while comparing recordings of voices.

## AUTHOR CONTRIBUTIONS

**Harriet M. J. Smith:** Conceptualization; investigation; writing – original draft; methodology; writing – review and editing; project administration; resources. **Kay L. Ritchie:** Conceptualization; investigation; writing – original draft; methodology; writing – review and editing; project administration; resources. **Thom S. Baguley:** Writing – review and editing; formal analysis; validation; software. **Nadine Lavan:** Writing – original draft; formal analysis; conceptualization; investigation; methodology; writing – review and editing; project administration; data curation; software; resources; visualization.

## CONFLICT OF INTEREST STATEMENT

All authors declare no conflict of interest.

## Supporting information


Data S1.

## Data Availability

Data to be made available upon publication at https://doi.org/10.17605/OSF.IO/WZCJU.

## Footnotes

^1^
Note that the majority of face‐matching literature focuses on simultaneous face‐matching, where both images are visible for comparison. In sequential face‐matching, there is a memory load as the second face must be compared to a representation of the first. However, when comparing only two images, overall accuracy is similar across simultaneous and sequential tasks (Menon et al., 2015; Ritchie et al., 2021).
