# Supplementary material for: Face and voice identity matching accuracy is not improved by multimodal identity information
Source: Br J Psychol. 2024 Dec 17;116(2):367–85. doi: 10.1111/bjop.12757 (PMC11984343; doi:10.1111/bjop.12757)
Supplement: Supplementary file 1 — Data S1. [file BJOP-116-367-s001.docx]

**Supplementary Analysis**

In addition to the analysis of the accuracy data, we below report on an analysis of our data using the signal detection measures D Prime and Criterion C.

For all analyses, we calculated D Prime and Criterion C following the guidelines provided in Stanislaw and Todorov (1999).

**Experiment 1**

We first ran a two-way ANOVA to assess whether having access to multimodal information during face matching and voice matching tasks improves D Prime. In this model, we included the matching task (face vs. voice) and other-stimulus modality (multimodal facevoice vs. unimodal face/unimodal voice) as factors. There was no interaction (*F*(1,195) < 0.01, *p* = 0.926) and no main effect of other-stimulus modality (F(1,195) = 0.64, p = 0.425). There was a main effect of modality (*F*(1,195) = 124.76, *p* < 0.001), with D Prime being higher for face matching than for voice matching. Data are plotted in Supplementary Figure 1 a).

We then ran the same model but now with Criterion C as a dependent variable. There was again no interaction (*F*(1,195) = 1.98, *p* = 0.161) and no main effect of other-stimulus modality (*F*(1,195) = 2.51, *p* = 0.115). There was, however, again a main effect of modality (*F*(1,195) = 8.27, *p* = 0.005), with Criterion being higher for face matching than for voice matching. Data are plotted in Supplementary Figure 1 b).

One-sample t-tests of condition-wise against 0 further shed light on the nature of this main effect: While Criterion C for multimodal face matching was no significantly different from 0 (*M* = 0.06; *SD* = 0.41; *t*(48) = 0.98, *p* = 0.331), it was significantly below 0 for unimodal face matching (*M* = -0.10; *SD* = 0.36; *t*(48 = -2.16, *p* = .049. Criterion C was also significantly below 0 for multimodal voice matching (*M* = -0.17; *SD*, = 0.38; *t*(49) = -.3.23, *p* = 0.002) and unimodal voice matching (*M* = -0.18, *SD* = 0.36; *t*(49) = -.3.59, *p* = 0.001). These results suggest that while participants were consistently biased towards responding “different identities” for voice matching, these effects were smaller unimodal face matching and entirely absent multimodal face matching. They furthermore confirm that response biases towards perceiving stimuli as belonging to different identities are more pronounced in the unimodal conditions compared to the multimodal conditions, underlining the main effect observed in the ANOVA.

*Supplementary Figure 1 a) D Prime and b) Criterion for face and voice matching when multimodal (for face-facevoice and voice-facevoice matching) or only unimodal information (for face-face and voice-voice matching) is available*


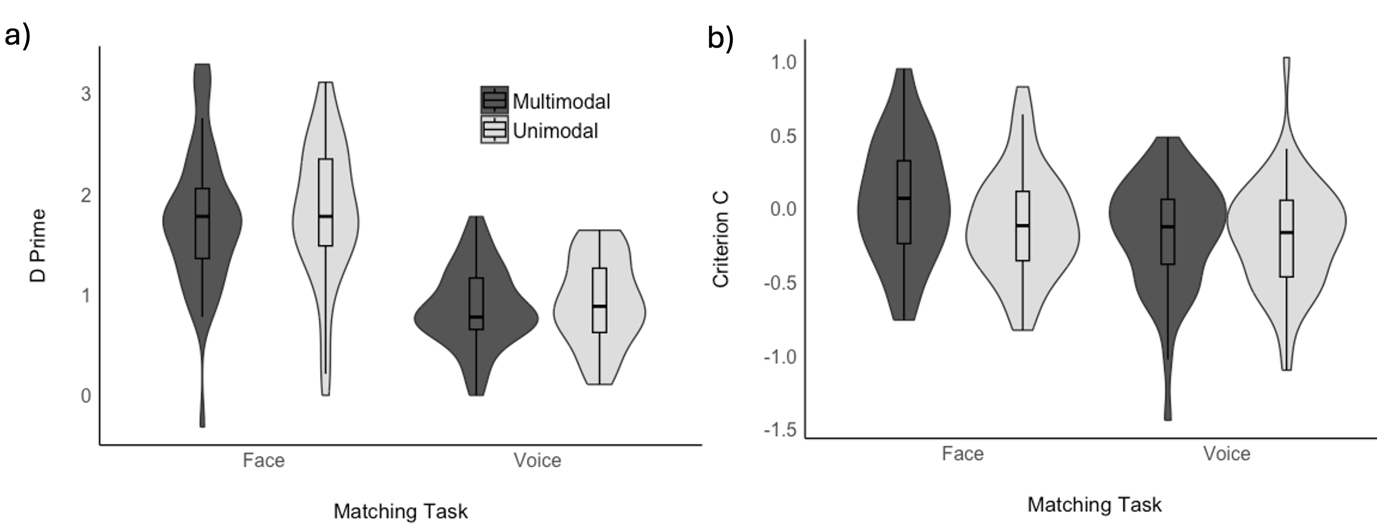


**Experiment 2**

As in the main manuscript, we now compare the face-face matching condition from Experiment 1 to the new facevoice-facevoice data from Experiment 1. To test whether having access to multimodal information for both stimuli in the matching task significantly increases D Prime or affects Criterion C compared to face-face matching, we ran another one-way ANOVA including the matching task (face-face vs facevoice-facevoice) as the factor of interest.

As shown in Supplementary Figure 2a, there was no main effect of modality (*F*(1,98) = 0.29, *p* = 0.591), with D Prime similar for face-face and facevoice-facevoice matching. We ran the same analysis for Criterion C and also find no main effect of matching task (*F*(1,98) = 0.943, *p* = 0.334) (see Supplementary Figure 2b). One-sample t-tests of Criterion against 0 further show that C is not significantly different from 0 for the facevoice-facevoice matching task (*t*(50) = -.36, = 0.720), further confirming that there is no biases in the responses for this condition.

*Supplementary Figure 2 a) D Prime and b) Criterion for face-face and facevoice-facevoice identity matching*


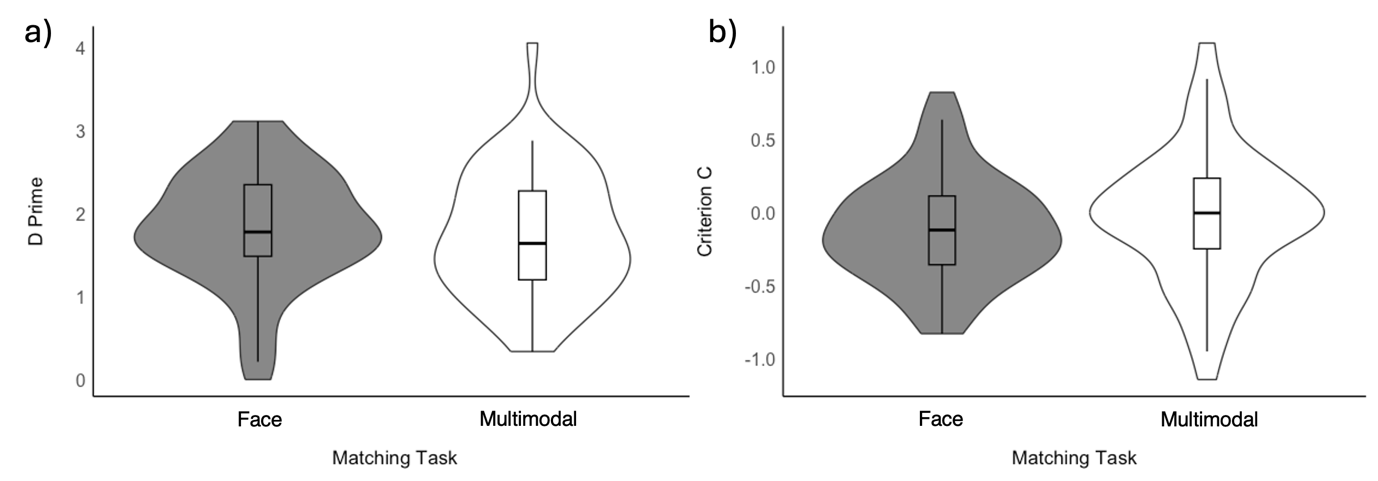


**Experiment 3**

*Supplementary Figure 3 a) D Prime and b) Criterion for crossmodal face-voice matching*


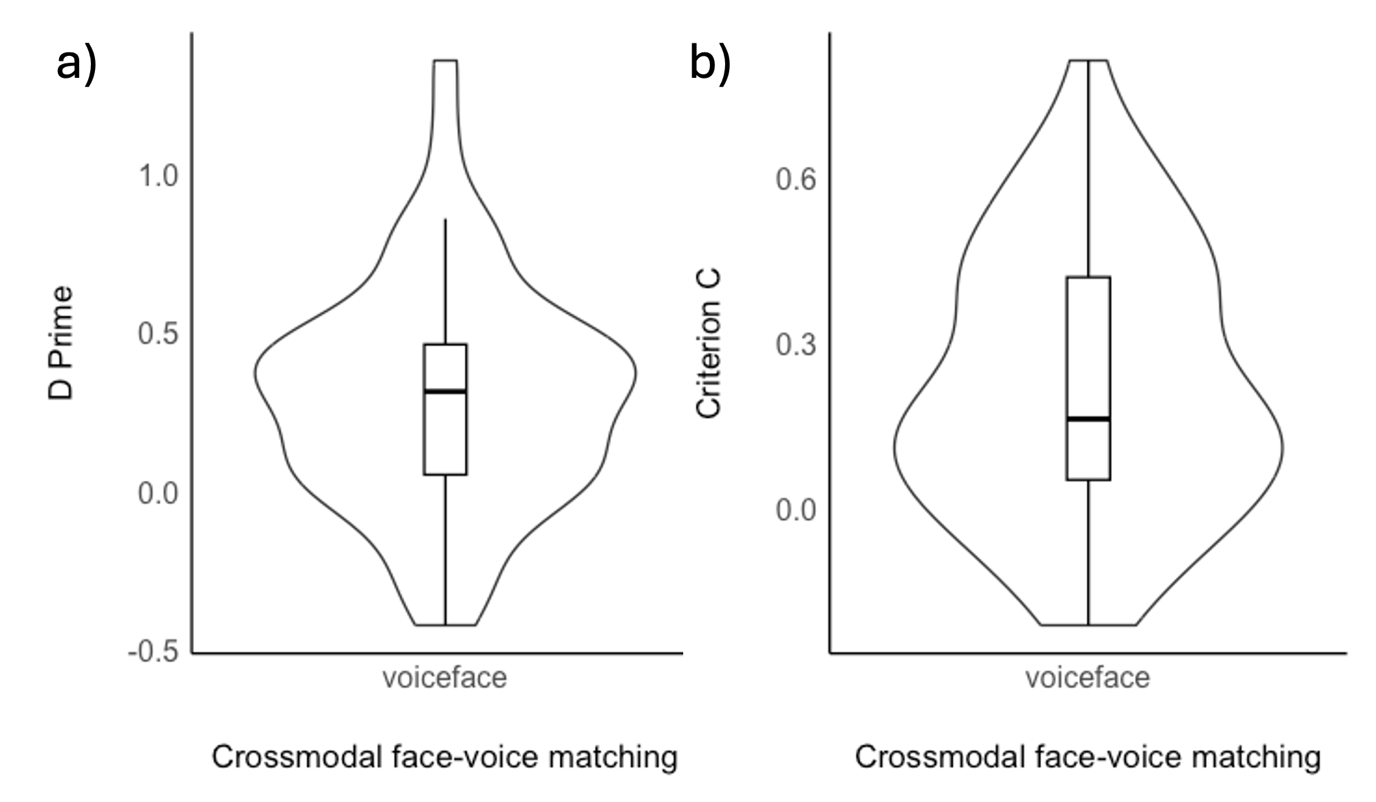


To test whether D Prime (Supplemetary Figure 3a) and Criterion C (Supplementary Figure 3b) significantly differ from 0 for crossmodal face-voice matching, we ran one-sample t-tests. These t-tests showed that both D Prime (*t*(50) = 6.69, *p* < .001) and Criterion C (*t*(50) = 5.97, *p* < .001) were larger than 0. This confirms the observation of a liberal response pattern for this task, where participants are biased towards perceiving faces and voices belonging to the same identity. D Prime was also significantly above 0 (*M* = 0.30, *SD* = 0.36) but also substantially lower than for any of the other conditions.
